# Work-related musculoskeletal disorders and ergonomic risk factors in special education teachers and teacher’s aides

**DOI:** 10.1186/s12889-016-2777-7

**Published:** 2016-02-10

**Authors:** Hsin-Yi Kathy Cheng, Man-Ting Wong, Yu-Chung Yu, Yan-Ying Ju

**Affiliations:** Graduate Institute of Early Intervention, College of Medicine, Chang Gung University, 259 Wen-Hua 1st Rd, Kwei-Shan, Tao-Yuan, 333 Taiwan; National Taoyuan Special School, Tao-Yuan, 330 Taiwan; Department of Adapted Physical Education, National Taiwan Sport University, 250 Wen-Hua 1st Rd, Kwei-Shan, Tao-Yuan, 333 Taiwan

**Keywords:** Work-related musculoskeletal disorders, Ergonomics, Special education, Teacher

## Abstract

**Background:**

Work-related musculoskeletal disorders (WMSDs) have become increasingly common among health-related professionals. Special education personnel who serve students with disabilities often experience physical strains; however, WMSDs have been overlooked in this population. The objectives of this study were to investigate the work-related ergonomics-associated factors in this population and to evaluate their correlation with the WMSDs prevalence.

**Methods:**

A questionnaire with three domains, namely demographics, prevalence of work-related musculoskeletal disorders, and ergonomic factors, designed by our research team was delivered to educators who work in special education schools.

**Results:**

Approximately 86 % of the 388 special education school teachers and teacher’s aides in this study experienced musculoskeletal disorders. The lower back, shoulder, and wrist were the three most affected regions. A logistic regression analysis revealed that the participants’ background factors, namely >5.5 years of experience (odds ratio [OR] = 4.090, 95 % CI: 1.350-12.390), students with multiple disorders (OR = 2.412, 95 % CI: 1.100-5.287), and other work-related ergonomic factors (assistance in diaper changing and others duties), were strongly associated with the prevalence of WMSD. Nap habit (OR = 0.442, 95 % CI: 0.230-0.851) and having teaching partners in the same class (OR = 0.486, 95 % CI: 0.250-0.945) resulted in low possibility of acquiring WMSDs. The use of supportive devices was associated with a low WMSD prevalence.

**Conclusions:**

The present study revealed an association between WMSDs and specific job features among teachers and teacher’s aides in special education schools. Future efforts should emphasize examining safe student-handling ergonomics, formulating policies regarding student-teacher ratio, incorporating mandatory break times at the workplaces, and promoting personal health for preventing work-related injuries.

**Electronic supplementary material:**

The online version of this article (doi:10.1186/s12889-016-2777-7) contains supplementary material, which is available to authorized users.

## Background

Apart from regular teaching, nursing care is a substantial part of the work content of teachers and teacher’s aides at special education schools for physically and mentally disabled students. Students at such schools mostly do not have the ability to control themselves, hold coordination, or adapt themselves [1]; therefore, teachers in these schools are responsible for meeting the daily needs of such students. The physical demands include but are not limited to frequently lifting and carrying students, transferring students from one place to another, assisting in positioning, changing diaper, feeding, prolonged standing, and pushing and pulling [2]. Because these students have delayed development, more assistance is required in their daily routines. In addition, students in special education schools tend to be overweight and exhibit abnormal muscle tone compared with their typically developing peers. Consequently, special education teachers and teacher’s aides are prone to musculoskeletal disorders.

Work-related musculoskeletal disorders (WMSDs) are defined as a subset of musculoskeletal disorders that arise from occupational exposures. WMSDs include a wide range of inflammatory and degenerative conditions affecting the musculoskeletal systems and can occur from a single traumatic event or cumulative overuse injuries [3]. This group of disorders represents one of the most common occupational health problems in the working population. WMSDs can affect the quality of life, resulting in increased sick leaves or early retirement and can impose a major economic burden because of compensation costs and lost wages [2].

Researchers have systematically reviewed WMSD prevalence among school teachers [3]. The prevalence of self-reported WMSDs among school teachers ranges between 39 % and 95 %, with the back, neck, and upper limbs being the most prevalent regions of symptoms. A teacher’s work includes teaching students, writing on the blackboard, preparing lessons, grading assignments, and school administrative work, which can cause adverse mental and physical health concerns [4–6]. In addition, sex, age, length of employment, and awkward postures are associated with a high prevalence of WMSDs.

Teachers who work in special education schools have not only teaching but also nursing care duties. They usually perform activities that require sustained periods of kneeling, stooping, squatting, bending, and constant trunk flexion [4, 7, 8]. Most studies investigating WMSDs among special education school teachers have focused on pain in a single specific body region, such as the lower back, and its associated factors [8, 9], and have concluded that the lower back pain is associated with a long-time static trunk flexion posture. Assistance in transferring, toileting, and feeding are also leading causes of lower back pain. Despite this, the prevalence of WMSDs within the special education profession has not received sufficient attention, and few studies have investigated WMSD prevalence and the possible associated human factors and ergonomics. Therefore, we investigated WMSD prevalence for clarifying the associated factors among special education teachers and teacher’s aides.

## Methods

### Instrumentation

A questionnaire was designed on the basis of the job specifics of special education teachers and teacher’s aides (WMSD questionnaire, see Additional file 1). The contents of this self-administered questionnaire were constructed and modified by reviewing relevant studies investigating WMSDs among school teachers and childcare workers. Four professors specializing in the field of special education, rehabilitation science, and early intervention reviewed the list of questions and suggested necessary modifications. A consensus was reached by these four experts, with a content validity index of 0.95. Interrater agreement (IRA) was also assessed for each item. The average IRA for the scale was 0.95. The final questionnaire included three domains: personal and institutional data, information regarding present WMSDs, and work-related ergonomic factors. The first domain was designed for obtaining personal information (sex, age, marital status, child-bearing history, body mass index [BMI], and academic major), work-related information (years of experience in special school, work days per week, work hours per day, break between lessons, nap habit, exercise habit, type of special school service provided, age-range of student served, diagnosis of students, teaching partners in the same class, supportive device usage while working, and feeling of stress), and information regarding past WMSDs (injury history, injury regions, duration since onset, treatment history, and relapse situation). The second domain captured information on WMSDs, including musculoskeletal injury prevalence based on the injury regions, severity, duration since onset, duration of symptoms, frequency of prevalence, causing sick leaves, considering a job change, affecting work performance, and participation in related continuing education courses. The design of this domain was in accordance with the Standardized Nordic Musculoskeletal Questionnaire [10]. The term “musculoskeletal disorders” here refers to work-related injuries that have occurred at any time during teachers’ work hours, lasted one or more days, and affected daily activities in the last 6 months. The investigation covered nine body regions: neck, shoulder, upper back, elbow, hand and wrist, lower back, thigh, knee, and ankle and foot. The pain resulting from the symptoms ranged from 0 (no pain) to 4 (unbearable pain). Participants responding with scores ≥1 were considered as experiencing WMSDs. The third domain involved information regarding work-related ergonomic factors (assistance in diaper changing, feeding, toileting, grooming, transferring, rehabilitation, and transporting) and the body regions with symptoms. Moreover, information on the requirement of improvements in the current job, namely work environment modifications, knowledge regarding the use of personal protective equipment, postural education, work-time adjustment, and muscle strengthening was collected.

For construct validity, 62 teachers from two special education schools were included in our survey. The Cronbach’s alpha coefficients for the questionnaire and WMSDs and ergonomic domains were 0.912, 0.916, and 0.885, respectively. All 62 participants filled the questionnaire again after two weeks, and test–retest reliability of 0.78 was reported.

### Sample and procedure

This study surveyed special education teachers and teacher’s aides working in special education schools for at least half a year. Those who had musculoskeletal injuries within the preceding six months caused by sources other than the workplace were excluded. Data from the Special Education Transmit Net, Ministry of Education (2014), indicated that there were 29 private and public special education schools in Taiwan, which employed 1882 teachers. A stratified sampling method was used for determining the number of special education schools included in this survey based on their geographical distribution. Twenty-one special education schools were selected through purposive sampling, and 588 teachers and teacher’s aides were surveyed.

The survey was conducted during the summer break to avoid interference in the school routine. The questionnaire with a cover letter explaining the purposes and procedure of the study were mailed to the participants. Those who agreed to participate provided their signatures as informed consents. Two weeks after sending the mails, the research team contacted those who had not returned filled questionnaires and encouraged them to respond to the survey. Meanwhile, the research team answered participant questions regarding participating in our study. This study procedure was approved by the Institutional Review Board of Chang Gung Memorial Hospital, who waived the requirement of written informed consents as this research involved no to minimal risks to the participants.

### Data processing and analysis

All analyses were conducted using the Statistical Package for Social Sciences 17.0 (SPSS Inc., Chicago, IL, USA). Descriptive statistics were performed to reveal the response distribution for each question in the domain capturing participants’ personal and institutional variables and for questions from the WMSD and ergonomics domains. Additional analyses were preformed using the *t* test and analyses of variance for the variables of personal and institutional data and ergonomics for evaluating the associations between these variables and the WMSD prevalence. A post hoc Tukey test was performed if statistically significant differences existed. The level of significance was set to 0.05.

**Fig. 1:**
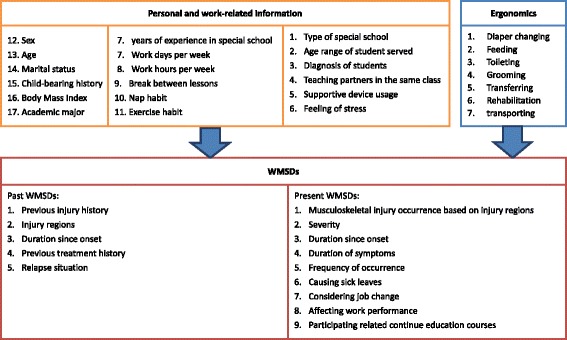
Research framework

Odds ratios (ORs) and 95 % confidence intervals (CIs) were calculated for examining the association between WMSDs and the variables from the personal and institutional data and ergonomics domain using a binary logistic regression. ORs were obtained for each potential factor after adjustment for age (categorical variable with five levels: <20, 21–30, 31–40, 41–50, and >50 years) and sex. The research framework depicting the variables and the associations to be tested are presented in Fig. 1. OR ≥ 1 was considered a contributor toward WMSDs, whereas OR < 1 was considered a protective factor.

## Results

### Participant demographics

Out of the 588 mailed questionnaires, 391 were returned, yielding a response rate of 70.1 %. One of the investigators requested for responses for incomplete sections over a telephonic conversation. Three questionnaires were excluded because the participants failed our inclusion criteria, which yielded 388 valid questionnaires. Among the valid questionnaires, 84.0 % (*n* = 326) were completed by female participants, most of whom were in the age group of 31–40 years (24.2 %, *n* = 94) and 45–50 years (44.8 %, *n* = 174). Most of the participants were married (71.4 %, *n* = 277) and had children (64.9 %, *n* = 252). Their average height and weight were 160.21 ± 6.52 cm and 59.19 ± 9.95 kg, respectively. The average BMI was 23.03 ± 3.32 kg/m^2^, which was within the normal range. Academically, 6.4 % (*n* = 25) of them majored in the field of special education, 15.5 % (*n* = 60) were in the field of childcare, 10.6 % (*n* = 41) were in the field of nursing or therapy, and 67.5 % (*n* = 262) majored with degrees other than those mentioned. Additional work-related information is presented in Table 1.

**Table 1:** Work profiles of special education teachers and teacher’s aides (*N* = 388)

Variables	Items	*N *(%)
Years of experience in SS^a^	<9 months	18 (4.6 %)
	9-18 months	28 (7.2 %)
	19-42 months	59 (15.2 %)
	43-66 months	43 (11.1 %)
	>66 months	240 (61.9 %)
Work days per week	<5 days	31 (8.0 %)
	5 days	308 (79.4 %)
	>5 days	49 (12.6 %)
Hours per day -	≤20 h	249 (64.2 %)
Administrative work	21-30 h	7 (1.8 %)
	31-40 h	53 (13.7 %)
	41-50 h	79 (20.4 %)
Hours per day -	≤20 h	104 (26.8 %)
Direct contact with students	21-30 h	12 (3.1 %)
	31-40 h	119 (30.7 %)
	41-50 h	153 (39.4 %)
Break between lessons	Yes	128 (33.0 %)
	No	260 (67.0 %)
Nap habit	Yes	167 (43.0 %)
	No	221 (57.0 %)
Exercise habit	Yes	200 (51.5 %)
	No	188 (48.5 %)
Type of special school	Public	388 (100 %)
	Subsidized	0 (0.0 %)
	Private	0 (0.0 %)
Age range of student served	Kindergarten	15 (4.1 %)
	Elementary school	42 (11.4 %)
	Secondary school	50 (13.6 %)
	Vocational School	192 (52.0 %)
	Dormitory	41 (11.1 %)
	Others	3 (0.8 %)
	More than one segment	26 (7.0 %)
Diagnosis of students	Physical disabilities	320 (85.6 %)
	Multiple disabilities	326 (87.2 %)
	Chromosomal abnormalities	134 (35.8 %)
	Hearing disabilities	120 (32.1 %)
	ASD^b^	314 (84.0 %)
	Rare disorder	143 (38.2 %)
	Intellectual disability	287 (76.7 %)
	Visual disabilities	111 (29.7 %)
	Others	22 (5.9 %)
Teaching partners in the same class	Yes	118 (30.4 %)
	No	270 (69.6 %)
Supportive device usage	upper extremity - Yes	64 (19.1 %)
	- No	94 (28.1 %)
	lower extremity - Yes	34 (8.8 %)
	- No	124 (32.0 %)
	Low back - Yes	121 (35.5 %)
	- No	37 (10.9 %)

^a^ *SS* special schools

^b^ *ASD* autism spectrum disorders

### Work-related musculoskeletal disorder information

#### Prevalence

In total, 333 special education teachers and teacher’s aides reported WMSDs symptoms, indicating a 85.8 % prevalence rate. WMSD severity was categorized into five levels from no pain (level 0) to unbearable pain (level 4). Participants were included in the analysis if they responded a severity level ≥1 in at least one musculoskeletal region considered in this study. High WMSDs prevalence rates of 68.8 % (*n* = 267), 63.4 % (*n* = 246), and 56.7 % (*n* = 220) were observed in the lower back, shoulder, and wrist, respectively, indicating presence of pain in these respective regions. Data on the variables, such as the duration since onset, duration of symptoms, and frequency of prevalence, for the aforementioned three regions are presented in Table 2.

**Table 2:** WMSDs characteristics in special education teachers and teacher’s aides (*N* = 388)

Variables	*N* (%)			
	Lower back	Shoulder	Wrist	Total count
Regional WMSDs occurrence (regional occurrence/388)	267 (68.8 %)	246 (63.4 %)	220 (56.7 %)	
Severity (pain)				
No pain	121 (31.2 %)	142 (36.6 %)	168 (43.3 %)	
Mild pain	88 (22.7 %)	100 (25.8 %)	118 (30.4 %)	
Moderate pain	119 (30.7 %)	113 (29.1 %)	65 (16.8 %)	
Severe pain	57 (14.7 %)	29 (7.5 %)	37 (9.5 %)	
Unbearable pain	3 (0.8 %)	4 (1.0 %)	0 (0 %)	
Duration since onset				
1 m to 3 m	69 (17.8 %)	53 (13.7 %)	85 (21.9 %)	
3 m to 5 m	35 (9.0 %)	24 (6.2 %)	33 (8.5 %)	
0.5 y to 1 y	45 (11.6 %)	30 (7.7 %)	46 (11.9 %)	
>1 year	72 (18.6 %)	76 (19.6 %)	79 (20.4 %)	
No symptoms	137 (35.3 %)	139 (35.8 %)	117 (30.2 %)	
Missing Data	30 (7.7 %)	66 (17.0 %)	28 (7.2 %)	
Duration of symptoms				
>1 month	61 (15.7 %)	53 (13.7 %)	53 (13.7 %)	
>3 months	27 (7.0 %)	24 (6.2 %)	18 (4.6 %)	
>6 months	18 (4.6 %)	30 (7.7 %)	23 (5.9 %)	
>1 year	95 (24.5 %)	76 (19.6 %)	64 (16.5 %)	
No symptoms	119 (30.7 %)	139 (35.8 %)	165 (42.5 %)	
Missing data	68 (17.5)	66 (17.0 %)	65 (16.8 %)	
Frequency of occurrence				
Almost everyday	112 (28.9 %)	98 (25.3 %)	84 (21.6 %)	
About once a week	55 (14.2 %)	52 (13.4 %)	43 (11.1 %)	
About twice a week	29 (7.5 %)	28 (7.2 %)	24 (6.2 %)	
About once a month	27 (7.0 %)	23 (5.9 %)	24 (6.2 %)	
No symptoms	122 (31.4 %)	138 (35.6 %)	167 (43.0 %)	
Missing data	42 (10.8 %)	49 (12.6 %)	46 (11.9 %)	
Causing sick leaves -Yes				60 (17.4 %)
-No				285 (82.6 %)
Considering job change-Yes				113 (33.4 %)
-No				225 (66.6 %)
Affecting work performance				
Not affecting				78 (20.1 %)
Mildly affecting				177 (45.6 %)
Moderately affecting				110 (28.4 %)
Severely affecting				19 (4.9 %)
Unable to work				4 (1.0 %)
Participating related continue education courses-Yes				216 (60.3 %)
-No				142 (39.7 %)

The lower back (32.7 %, *n* = 127), shoulder and wrist (10.6 %, *n* = 41), and knee (5.4 %, *n* = 21) were the most affected body regions among the participants, whereas and 17.8 % (n = 69) of the participants stated involvement of more than one body region. Data on sick leaves, thoughts of changing job, and other affected factors because of WMSDs are presented in Table 2.

#### Ergonomic factors-related information

The most frequently performed tasks included assistance in toileting (82.2 %, n = 319), grooming (80.7 %, *n* = 313), diaper changing (78.1 %, *n* = 303), feeding (76.3 %, *n* = 296), transferring (72.7 %, *n* = 282), rehabilitating (66.8 %, *n* = 259), and getting in and out of a vehicle (92 %, *n* = 293). The percentage of the participants reporting a requirement of improvements regarding postural education, knowledge in personal supportive device usage, muscle strengthening, work-time adjustment, and work environment modifications were 73.7 %, 72.6 %, 67.5 %, 63.2 % and 54.3 %, respectively.

#### Regression analysis for WMSDs prevalence

The results of the logistic regression model revealed that the length of employment (years of experience) produced an OR of 4.090 (95 % CI: 1.350–12.390), indicating that those who worked in special education schools for more than 66 months were 4.090 times more likely to have WMSDs than their colleagues who worked for a shorter duration. In addition, students diagnosed with multiple disorders produced an OR of 2.412 (95 % CI: 1.100–5.287), implying that those who took care of students with multiple disabilities were 2.412 times more likely to experience WMSDs than those who did not.

By contrast, the participants who had nap habit showed an OR of 0.442 (95 % CI: 0.230–0.851), indicating that they were 0.558 times less likely to experience WMSDs than their peers without nap habit. Those who had teaching partners in the same class also produced an OR of 0.486 (95 % CI: 0.250–0.945), indicating that those who have teaching partners are 0.514 times less likely to experience WMSDs than their peers who did not have teaching partners.

Furthermore, all work-related ergonomic factors demonstrated ORs with close proximity to 1. Teachers whose work did not involve assisting in diaper changing (OR = 0.986, 95 % CI: 0.976-0.996), feeding (OR = 0.986, 95 % CI: 0.976-0.997), toileting (OR = 0.987, 95 % CI: 0.976-0.997), grooming (OR = 0.987, 95 % CI: 0.976-0.997), transferring (OR = 0.987, 95 % CI: 0.976-0.997), rehabilitation (OR = 0.987, 95 % CI: 0.977-0.997), and getting in and out of a vehicle (OR = 0.988, 95 % CI: 0.978-0.998) were 0.012–0.014 times less likely to experience WMSDs than their peers. The summary of the logistic regression results is presented in Table 3.

**Table 3:** Logistic regression analysis: summary of results of personal/institutional data and ergonomic variables on WMSDs (*N* = 388)

Factors	B	SE	Wald	df	Significance	OR^a^	95 % CI^b^ for Exp (B)	
							Lower	Upper
Years of experience-over 66 months	1.409	.565	6.205	1	.013*	4.090	1.350	12.390
Nap habit-yes	−8.163E-01	.334	5.959	1	.015*	.442	.230	.851
Diagnosis of students-multiple disorders	.881	.400	4.836	1	.028*	2.412	1.100	5.287
Teaching partners in the same class-yes	−7.206E-01	.339	4.521	1	.033*	.486	.250	.945
Diaper changing-no	-.014	.005	6.985	1	.008**	.986	.976	.996
Assist feeding-no	-.014	.005	6.854	1	.009**	.986	.976	.997
Assist toileting-no	-.013	.005	6.499	1	.011**	.987	.976	.997
Assist grooming-no	-.013	.005	6.514	1	.011**	.987	.976	.997
Assist transferring-no	-.013	.005	6.508	1	.011**	.987	.976	.997
Assist rehabilitation-no	-.013	.005	6.426	1	.011**	.987	.977	.997
Assist getting in/out vehicle-no	-.012	.005	5.558	1	.018**	.988	.978	.998

The OR was adjusted for age and sex

*p < 0.05, **p < 0.01

^a^ *OR* odds ratio

^b^ *CI* confidence interval

#### Correlation between pain in different body regions

Lower back pain was positively correlated to pain in the elbow, wrist, and knee (correlation coefficients [r] = 0.364, 0.377, and 0.412, respectively; *P* < 0.01). Shoulder pain was positively correlated to pain in the upper back, lower back, elbow, wrist, and knee (*r* = 0.530, 0.470, 0.454, and 0.481, respectively; *P* < 0.01). Furthermore, wrist pain was moderately correlated to pain in the neck, shoulder, upper back, lower back, and elbow (*r* = 0.525, 0.481, 0.490, 0.377, and 0.511, respectively; *P* < 0.01). These results indicated that pain in one region was associated with pain in the other regions, specifically for the regions which are anatomically close to each other.

#### Correlation between pain and supportive devices

Supportive device usage was positively correlated with its corresponding body regions. A back support or brace was significantly correlated with the lower back pain (*r* = 0.320, *P* < 0.01), a knee support or brace was significantly correlated to knee pain (*r* = 0.239, *P* < 0.01), and wrist and elbow supportive devices were significantly correlated with wrist and elbow pain (*r* = 0.260, 0.216, respectively; *P* < 0.01).

## Discussion

### Factors associated with WMSDs prevalence

Teaching physically and mentally disabled students is physically demanding. The reported WMSD prevalence among regular school teachers ranges between 39 % and 95 %. Our results showed that WMSD prevalence among special education school teachers and teacher’s aides was 85.8 %, implying that WMSDs occur in over eight out of 10 teachers and aides in the target population; therefore, attention must be given. Compared with regular school teachers, this rate was higher. Special education school teachers and teacher’s aides, compared with regular school teachers, spend a substantial portion of their work-days in tasks involving movements and postures, which stress their bodies [2]. They face more challenges in tending to the students with special needs. These students have difficulties taking care of themselves. In addition, the majority of the students in our study were diagnosed with multiple disabilities (84.0 %, *n* = 326), which required multidimensional attention, consequently increasing teachers’ workload and reducing the rest period [8]. The results indicated that teachers who tended to students with multiple disorders were more likely to experience WMSDs than were those who did not.

Length of employment (years of experience) influenced the WMSD prevalence. Teachers employed for more than 66 months were more likely to acquire WMSDs compared with their colleagues who worked for a shorter period. Based on the qualitative feedbacks from the participants, experienced teachers and teacher’s aides were usually assigned to tend to difficult students; a few of the teachers and aides developed microtrauma from daily caregiving duties, which accumulated over time. The persisting, repetitive stress and work overload intensify the problems.

By contrast, the participants who had teaching partners in the same class or had a nap habit had lower chances of experiencing WMSDs. Teaching partners in the same class share workloads, resulting in a reduced possibility of acquiring WMSDs [2]. Da Costa and Vieira [11] indicated that a high biomechanical demand was one of the factors that caused WMSDs among their study participants. It is reasonable to assume that participants who had helpers in the same class and those who took naps during the working hours reduced few of the physical demands, which allowing their bodies to recover. Therefore, these factors were related to a low WMSDs prevalence rate.

Furthermore, participants who engaged in additional students’ daily caregiving duties were more likely to acquire WMSDs. Generally, students with multiple disabilities are highly dependent, and caregivers must provide great assistance in their daily activities, which includes assistance in diaper changing, feeding, toileting, grooming, transferring, rehabilitation, and getting in and out of vehicles. These duties impact their bodies considerably, especially the musculoskeletal systems. This finding is consistent with the findings of a study in Japan, where 72 % of the investigated special education school teachers serving physically and mentally disabled students reported upper limb WMSDs [12].

### Factors related to regional WMSDs

In our study, the most affected regions were the lower back, shoulder, and wrist, in that order; this is similar to the ranking reported in Vedovato and Monteiro [13] and Muto et al. [9], which involved school teachers, and Gratz and Claffey [14], which involved childcare workers. Moreover, our data indicated that pain in certain regions was positively associated with pain in other regions. In this study, lower back pain was positively correlated with pain in the elbow, wrist, and knee; shoulder pain was positively correlated with pain in the upper back, lower back, elbow, and wrist; and wrist pain was moderately correlated with pain in the neck, shoulder, upper back, lower back, and elbow. Anatomically, the shoulder and upper back, elbow, and wrist are together considered upper quadrant. During upper extremity–involving activities, such as carrying and feeding, these body parts work together. While carrying students, the back functions as a stabilizer, whereas the neck, shoulder, elbow, and wrist work together as the primary mover. Considering these biomechanical links, pain in one of these regions inevitably imposes extra loads on the others, inducing pain in the other regions.

### Factors associated with supportive device usage

Our results show that a low WMSD prevalence was associated with the use of supportive devices. The use of a lower back support or brace was associated with a reduced lower back pain rate. Similar trend applied to the usage of knee supportive device or brace and wrist and elbow supportive devices. Over 80 % of our participants wore supportive devices as preventive measures for possible work-related injuries. The qualitative feedbacks indicated that few schools provided supportive devices to their staffs for preventing WMSDs and for avoiding reimbursement of work-related injury claims.

### Qualitative feedbacks from the participants

Our participants also provided important suggestions and commands, which require serious attention. First, most participants suggested that courses, such as postural education, should be held on a regular basis. Most of their work duties included lifting and transferring, which are frequently related to the lower back pain [2, 8, 9, 15]. Knowledge regarding positioning, proper method of lifting, and muscle strengthening would enhance safer body biomechanics, thus preventing injuries such as muscle strain or ligament sprain [2, 16].

Secondly, sharing workload is also an essential factor. Proper allocation of manpower greatly reduces the WMSD prevalence and improves the work and caregiving qualities. In addition, participants who worked more for than five and a half years demonstrated significantly higher risk of acquiring WMSDs. Overuse injuries are often caused by repetitive usage of certain body parts. A single force may not result in injury; however, repetition, energy, and accumulated microtrauma can lead to WMSDs.

The health of teachers and teacher’s aides are essential not only for their own benefits but also for providing superior care to the students. Our results are an alert for the relevant units, such as the education department, to pay more attention to the health concerns of special education workers.

### Limitations

There are a few limitations to the present study. First, 30 % of the participants did not respond. Because the authors did not verify whether the participants who did not respond differed from those who responded, the results may have a selection bias. Second, this study only excluded participants who had musculoskeletal injuries, such as caused by sources other than the workplace within the past half year. All other participants, including those who experienced only mild pain or even numbness without affecting daily activities, were included, which may have resulted in the high prevalence rate of WMSDs observed in our study compared with other related studies. Third, non–work-related activities were not controlled for or accounted for the inclusion or exclusion criteria. Non–work-related activities should have been included in the present study as confounding factors. Fourth, the survey was conducted during the summer break, which may have underestimated the prevalence rate of WMSDs because the school year is usually more stressful. Finally, this study relied on self-reported WMSDs, which is inevitably subject to recall bias, which could have revealed participants’ subjective perception of WMSDs during the survey. Our results should be interpreted with caution. The aforementioned limitations can be overcome through further research.

## Conclusions

In the present study, we surveyed WMSD prevalence among special education school teachers and teacher’s aides. Our results indicated a high prevalence rate of 85.8 % and a strong association between WMSDs and teachers’ personal backgrounds and work-related ergonomic factors. The lower back, shoulder, and wrist were the three most affected regions. WMSD prevalence increased along with the presence of students with multiple disabilities in the classroom. Furthermore, the use of supportive devices was associated with a low WMSD prevalence. The presented results provide associated professional organizations general insights to WMSD prevalence among special education school teachers and teacher’s aids. The results are expected to focus more attention on career-related health concerns. The findings serve as guidelines to special education schools for modifying work environments, planning education courses, and making other related changes for reducing WMSD prevalence. The results of this study provide guidance to special education school teachers and teacher’s aids regarding safe student-handling ergonomics and health awareness. Further research should emphasize the analysis of specifics regarding teacher’s job features, such as the type and severity of the students’ disabilities and the teacher–student ratio in a class, and the association between these job features and the prevalence and prevention of WMSDs.

## Additional file

Additional file 1:
**WMSD questionnaire**. (DOCX 51 kb)
