# Supplementary material for: Work-related musculoskeletal disorders and ergonomic risk factors in special education teachers and teacher’s aides
Source: BMC Public Health. 2016 Feb 10;16:137. doi: 10.1186/s12889-016-2777-7 (PMC4750223; doi:10.1186/s12889-016-2777-7)
Supplement: Additional file 1: — WMSD questionnaire. (DOCX 51 kb) [file 12889_2016_2777_MOESM1_ESM.docx]

**Appendix - Investigation for Work-related Musculoskeletal Disorders**

|  |
| --- |

| **1. Backgrounds** | | | | | | | | | | | | | | | | | | | | | | | | | | | | | | | | |
| --- | --- | --- | --- | --- | --- | --- | --- | --- | --- | --- | --- | --- | --- | --- | --- | --- | --- | --- | --- | --- | --- | --- | --- | --- | --- | --- | --- | --- | --- | --- | --- | --- |
| **Note: check the appropriate box** | | | | | | | | | | | | | | | | | | | | | | | | | | | | | | | | |
| 1. | **Sex** | **□Male** | | **□Female** | | | | |  | | | |  | | | | | | | | | |  | | | | | | | | | |
| 2. | **Age** | **□≦20 years** | | **□21-30years** | | | **□31-40years** | | | | | | **□41-50years** | | | | | | | | | | **□>50years** | | | | | | | | | |
| 3 | **Marital status** | **□Unmarried** | | **□Married** | | | **□Separate** | | | | | | **□Divorced** | | | | | | | | | | **□Widow/widower** | | | | | | | | | |
| 4 | **Child-bearing history**  **(female only)** | | | **□No** | | | **□Yes** | | | | | | **Number of Children: __________** | | | | | | | | | | | | | | | | | | | |
| 5 | **Highest degree earned** | **□High school** | | **□College** | | | **□University** | | | | | | **□Graduate school** | | | | | | | | | | | | **□Others** | | | | | | | |
| 6 | **Academic major** | **□Special Edu/ Education** | | | | | **□Early Childhood Care / Child care** | | | | | | | | | | | | | | | | | | **□Others** | | | | | | | |
|  |  | **□Nursing / rehabilitation/ physical therapy/ occupational therapy/ speech therapy** | | | | | | | | | | | | | | | | | | | | | | | | | | | | | | |
| 7 | **Height** | **cm** | | | | | | | | | | | | | | | | | | | | | | |  | | | | | | | |
| 8 | **Weight** | **kg** | | | | | | | | | | | | | | | | | | | | | | |  | | | | | | | |
| 9 | **Work hours per week** | | **Administrative work**  **Childcare related tasks** | | | | | | | **□<20**  **□<20** | | | **□20-30**  **□20-30** | | | | | | **□30-40**  **□30-40** | | | | | | **□40-50**  **□40-50** | | | | | | | |
| 10 | **Work days per week** | | | | | | | | | **□<5** | | | **□5** | | | | | | **□>5** | | | | | |  | | | | | | |  |
| 11 | **Break time between class transition？** | | | | | | | | | **□No** | | | **□Yes, times/day   minutes/each time** | | | | | | | | | | | | | | | | | | | |
| 12 | **Nap habit?** | | | **□No** | | | | | | **□Yes, minutes/each time** | | | | | | | | | | | | | | | | | | | | | | |
| 13 | **Exercise habit** | | | **□No** | | | | | | **□Yes** | | | | | | | | | | | | | |  | | | | | |  | | |
|  | **Types of exercise: , frequency: _____times/week** | | | | | | | | | | | | | | | | | | | | | | | | | | | | | | | |
| 14 | **Name of the special school(s) you provide service to:** | | | | | | | | | | | | | | | | | | | | | | | | | | | | | | | |
| 15 | **Geometrical distribution of the special school(s)** | | | | **□North** | | | | | | | | | | | | | | | | | | | | | | | | | | | |
|  |  |  |  |  | **□Central** | | | | | | | | | | | | | | | | | | | | | | | | | | | |
|  |  |  |  |  | **□Southern** | | | | | | | | | | | | | | | | | | | | | | | | | | | |
|  |  |  |  |  | **□Eastern** | | | | | | | | | | | | | | | | | | | | | | | | | | | |
|  |  |  |  |  | **□off-shore islands** | | | | | | | | | | | | | | | | | | | | | | | | | | | |
| 16 | **School fund source** | | | | **□Public** | | | | | | | **□Public-private** | | | | | | | | | **□Private** | | | | | |  | | | | | |
| 17 | **Years of experience in SS^a^**  (after the first 6 month) | | | | **□<3 months** | | | | | | | **□3-12 months** | | | | | | | | | **□1-3 years** | | | | | | | **□3-5 years** | | | | |
|  |  |  |  |  | **□>5 years** | | | | | | |  | | | | | | | | |  | | | | | |  | | | | | |
| 18 | **Did you have WMSD^b^ in your last job?** | | | | **□No (jump to Q24)** | | | | | | | | | | | **□Yes** | | | | | | | | | | | | | | | | |
| 19 | **Which parts of the body suffered from WMSD in your last job?** | | | | **□ Neck** | | | **□Shoulder** | | | | | | **□Upper back** | | | | | | | | **□Lower back** | | | | | | | | **□Elbow** | | |
|  |  |  |  |  | **□Hands & Wrist** | | | | | | **□Thighs** | | | | | | **□Knee** | | | | | | | | | **□Feet & Ankles** | | | | | | |
| 20 | **How long did you suffer from WMSD in your last job?** | | | | **□One month** | | | | | **□Three months** | | | | | | | | **□Half year** | | | | | | | | | | | **□___months** | | | |
| 21 | **Did you seek for any treatment about WMSD in your last job?** | | | | **□No** | | | | | **□Yes, type of treatment: _______________________** | | | | | | | | | | | | | | | | | | | | | | |
| 22 | **Did you feel satisfied with the treatment?** | | | | **□Yes** | | | | | **□No** | | | | | | | | **□Not certain** | | | | | | | | | | |  | | | |
| 23 | **Did you suffer a relapsed of WMSD from the same place in the current job?** | | | | **□Yes** | | | | | **□No** | | | | | | | | **□Not certain** | | | | | | | | | | |  | | | |
| 24 | **Age range of students served** | | | | **□preschool □College** | | | | | **□elementary school**  **□others_______** | | | | | | | | **□junior high school** | | | | | | | | | | | **□high school** | | | |
| 25 | **No. of students served everyday** | | | | **□<12** | | | | | **□13-15** | | | | | | | | **□16-20** | | | | | | | | | | | **□>21** | | | |
| 26 | **Diagnoses of the students (multiple choice)** | | | | **□Physical**  **disabilities** | | | | | **□Multiple**  **disabilities** | | | | | | | | **□ADHD/ ADD** | | | | | | | | | | | **□Down**  **syndrome** | | | |
|  |  |  |  |  | **□ASD** | | | | | **□Rare disorders** | | | | | | | | **□Intellectual disability** | | | | | | | | | | | | | | |
|  |  |  |  |  | **□Others** | | | | | | | | | | | | | | | | | | | | | | | | | | | |
| 27 | **Teaching partners in same class: □Yes: # of teaching partners** | | | | | | | | | | | | | | | | | | | **□No** | | | | | | | | | | |  | |
| 28 | **Monthly salary (NTD)** | | **□ < 25,000** | | | **□ 25,000 ~ 30,000** | | | | | | | | | **□ 30,001 ~ 35,000** | | | | | | | | | | **□≧ 35,001** | | | | | | | |

**2. Musculoskeletal symptoms**

| **Note: check the appropriate box (multiple choice)** |
| --- |

29. **Any of the followings occurred within this half year?**

| **Region**  **Symptom** | | Neck | Shoulder | Upper  back | Lower back | Elbow | Hands  &  Wrist | Thighs | Knee | Feet  &  Ankles |
| --- | --- | --- | --- | --- | --- | --- | --- | --- | --- | --- |
| **Severity** | **No pain** |  |  |  |  |  |  |  |  |  |
|  | **Mild pain** |  |  |  |  |  |  |  |  |  |
|  | **Moderate**  **pain** |  |  |  |  |  |  |  |  |  |
|  | **Severe pain** |  |  |  |  |  |  |  |  |  |
|  | **Unbearable**  **pain** |  |  |  |  |  |  |  |  |  |
| **Duration**  **since onset** | **Not applicable** |  |  |  |  |  |  |  |  |  |
|  | **< 1month** |  |  |  |  |  |  |  |  |  |
|  | **1 month**  **– 0.5year** |  |  |  |  |  |  |  |  |  |
|  | **0.5 year**  **– 1 year** |  |  |  |  |  |  |  |  |  |
|  | **> 1 year** |  |  |  |  |  |  |  |  |  |
| **Duration of symptoms** | **Not applicable** |  |  |  |  |  |  |  |  |  |
|  | **More than 1 month** |  |  |  |  |  |  |  |  |  |
|  | **More than 3 months** |  |  |  |  |  |  |  |  |  |
|  | **More than 6 months** |  |  |  |  |  |  |  |  |  |
|  | **More than 1 year** |  |  |  |  |  |  |  |  |  |
| **Frequency of occurrence** | **Not applicable** |  |  |  |  |  |  |  |  |  |
|  | **Almost everyday** |  |  |  |  |  |  |  |  |  |
|  | **About once**  **a week** |  |  |  |  |  |  |  |  |  |
|  | **About once every two weeks** |  |  |  |  |  |  |  |  |  |
|  | **About once**  **a month** |  |  |  |  |  |  |  |  |  |

| 30 | **Check the most affected region (please tick only one box)** | | | | |
| --- | --- | --- | --- | --- | --- |
|  | **□Neck □Shoulder □Upper back □Lower back □Hand, Wrist □ Elbow**  **□Knee □Thigh □ Foot, Ankle** | | | | |
| 31 | **Affecting work performance** | **□Not affected** | | | |
|  |  | **□Mild affected** | | | |
|  |  | **□Moderately affected** | | | |
|  |  | **□Severely affected** | | | |
|  |  | **□Unable to work** | | | |
| 32 | **Causing sick leaves** | **□No** | **□Yes** |  |  |
| 33 | **Seeking treatment** | **□Ignored** | **□Massage** | **□ rehabilitation treatment** | **□ surgical intervention** |
|  |  | **□Medication**  **(oral)** | **□Medication**  **(topical)** | **□Heat /Cold therapy** | **□Other** |
| 34 | **Considering job change** | **□No** | **□Yes** |  |  |
| 35 | **Supportive device usage while working** | **□No** | **□Yes：**  **□wrist support □elbow support □knee support**  **□lumbar support □ankle brace □others** | | |
| 36 | **Participating related continuing education courses** | **□No** | **□Yes** |  | |

**3. Ergonomic factors**

| **Note: check the appropriate box (multiple choice)** |
| --- |

| Work content | **Regions with symptoms** | | | | | | | | | |
| --- | --- | --- | --- | --- | --- | --- | --- | --- | --- | --- |
|  | Task execution | Neck | Shoulder | Upper  back | Lower back | Elbow | Hand  &  Wrist | Thigh | Knee | Foot  &  Ankle |
| 37.diaper  changing | □Yes  □No |  |  |  |  |  |  |  |  |  |
| 38.feeding | □Yes  □No |  |  |  |  |  |  |  |  |  |
| 39.toileting | □Yes  □No |  |  |  |  |  |  |  |  |  |
| 40.grooming | □Yes  □No |  |  |  |  |  |  |  |  |  |
| 41.transferring | □Yes  □No |  |  |  |  |  |  |  |  |  |
| 42. rehabilitation | □Yes  □No |  |  |  |  |  |  |  |  |  |
| 43. transporting | □Yes  □No |  |  |  |  |  |  |  |  |  |

38. Ergonomic needs

|  | **0**  **Not needed** | **1**  **Not as needed** | **2**  **Neutral** | **3**  **Somewhat needed** | **4**  **Highly needed** |
| --- | --- | --- | --- | --- | --- |
| **Environmental modification**  (such as: size and height of tables, chairs, etc.) |  |  |  |  |  |
| **Knowledge regarding the use of personal protective equipment**  (Timing for the use of lumbar supports, knee braces, etc.) |  |  |  |  |  |
| **Postural education**  (proper body biomechanics, child handling techniques, etc.) |  |  |  |  |  |
| **Work-time adjustment**  (such as ways to arrange the work and break time) |  |  |  |  |  |
| **Muscle strengthening**  (frequency, intensity and duration) |  |  |  |  |  |

a: special school b: Work-related Musculoskeletal Disorders

**~Thank you for your participation~**
